# Home-based chlamydia and gonorrhoea screening: a systematic review of strategies and outcomes

**DOI:** 10.1186/1471-2458-13-189

**Published:** 2013-03-04

**Authors:** Muhammad S Jamil, Jane S Hocking, Heidi M Bauer, Hammad Ali, Handan Wand, Kirsty Smith, Jennifer Walker, Basil Donovan, John M Kaldor, Rebecca J Guy

**Affiliations:** 1 The Kirby Institute, University of New South Wales, Sydney, NSW, Australia; 2 Center for Women’s Health, Gender and Society, Melbourne School of Population Health, University of Melbourne, Melbourne, VIC, Australia; 3 Division of Epidemiology, School of Public Health, University of California, Berkeley, CA, USA; 4 Centre for Excellence in Rural Sexual Health, Rural Health Academic Centre, Melbourne Medical School, University of Melbourne, Melbourne, VIC, Australia; 5 Sydney Sexual Health Center, Sydney Hospital, Sydney, NSW, Australia

**Keywords:** Sexually transmitted infections, Chlamydia trachomatis, Screening, Home

## Abstract

**Background:**

In many countries, low *Chlamydia trachomatis* (CT) and *Neisseria gonorrhoeae* (NG) screening rates among young people in primary-care have encouraged screening programs outside of clinics. Nucleic acid amplification tests (NAATs) make it possible to screen people in homes with self-collected specimens. We systematically reviewed the strategies and outcomes of home-based CT/NG screening programs.

**Methods:**

Electronic databases were searched for home-based CT and/or NG screening studies published since January 2005. Screening information (e.g. target group, recruitment and specimen-collection method) and quantitative outcomes (e.g. number of participants, tests and positivity) were extracted. The screening programs were classified into seven groups on the basis of strategies used.

**Results:**

We found 29 eligible papers describing 32 home-based screening programs. In seven outreach programs, people were approached in their homes: a median of 97% participants provided specimens and 76% were tested overall (13717 tests). In seven programs, people were invited to receive postal test-kits (PTKs) at their homes: a median of 37% accepted PTKs, 79% returned specimens and 19% were tested (46225 tests). PTKs were sent along with invitation letters in five programs: a median of 33% returned specimens and 29% of those invited were tested (15126 tests). PTKs were requested through the internet or phone without invitations in four programs and a median of 32% returned specimens (2666 tests). Four programs involved study personnel directly inviting people to receive PTKs: a median of 46% accepted PTKs, 21% returned specimens and 9.1% were tested (341 tests). PTKs were picked-up from designated locations in three programs: a total of 6765 kits were picked-up and 1167 (17%) specimens were returned for screening. Two programs used a combination of above strategies (2395 tests) but the outcomes were not reported separately. The overall median CT positivity was 3.6% (inter-quartile range: 1.7-7.3%).

**Conclusions:**

A variety of strategies have been used in home-based CT/NG screening programs. The screening strategies and their feasibility in the local context need to be carefully considered to maximize the effectiveness of home-based screening programs.

## Background

*Chlamydia trachomatis* (CT) is the most common notifiable sexually transmissible infection (STI) in the United States (US) [1], Europe [2] and Australia [3]. Many countries have experienced substantial increase in reported CT infections over the past decade. Screening for CT and *Neisseria gonorrhoeae* (NG) is important because most infections remain asymptomatic and often undiagnosed [4,5]. Untreated infections can result in major sequelae including pelvic inflammatory disease, ectopic pregnancy, chronic pain, and infertility in women and epididymitis in men [5].

Clinical guidelines recommend annual CT screening for sexually active young women in many countries [6-8], and also sexually active men in some countries [9]. For NG screening, local prevalence and individual risk factors should be considered [6,8]. Opportunistic screening of people attending primary-care clinics for non-sexual health reasons has usually failed to achieve high coverage [10-12]. This may be due to practitioner reported barriers including lack of knowledge of the benefits of screening, concerns about upsetting patients, time constraints, lack of reminder systems and little support for contact tracing [13,14]. Low attendance rates for routine care among many at-risk people, particularly young men, also play a role in low screening rates [15-17].

The advent of nucleic acid amplification tests (NAATs) has made it possible to screen people in homes with self-collected specimens. Home-based screening, with urine or self-collected vaginal specimens, has been shown to be acceptable and has the potential to reach people who do not get tested otherwise [18]. A recent review reported that home-based STI screening resulted in up to 11 times higher testing rates compared to the clinic-based screening [19]. A randomized control trial (RCT) showed that 83% of women in a home-based CT screening arm indicated a preference for future home-screening compared to 49% in the clinic arm who preferred future screening in clinics [20]. We conducted a systematic review of published literature on home-based CT and NG screening to explore the strategies used for screening and the key outcomes of screening programs including participation rates, testing rates, treatment rates and the positivity.

## Methods

This systematic review was conducted according to the PRISMA guidelines [21].

### Search strategy

The electronic bibliographic databases, PubMed and EMBASE, were searched for English language studies published between January 1, 2005 and January 28, 2011 with the search terms: Chlamydia, or Chlamydia infections, or Chlamydia trachomatis, OR Gonorrhea, AND Screening, or Mass Screening, or testing. The search was restricted to 2005 onwards, since most programs involving home-based screening have been established in recent years. The reference lists of selected studies were screened for other potentially relevant studies.

### Inclusion criteria

Papers were reviewed by two authors independently and disagreements were resolved by discussion and consensus. A study was included if it described a CT or CT and NG screening program with self-collected specimens at home and reported the number of tests. For studies in more than one setting, only home-based screening data were included. RCTs were included, with the data from home-testing arm only.

Studies were excluded if: no original data was reported, such as reviews or editorials; screening was conducted in both clinics and home but home-screening data were not reported separately; screening was conducted as part of a cohort study as the testing rates would be falsely elevated; or screening was anonymous where the test results could not be provided back to individuals.

### Data extraction and analysis

One author extracted the data from each paper and a second author verified the data. The following information was extracted: demographics; recruitment strategy (target group, advertisement, reminders); specimens collected; test-kit and specimen delivery method; incentives provided; number of people invited, participated and screened; CT/NG positivity; notification of results and treatment; and the cost of tests (also converted to US dollars for comparison). The authors were contacted to collect additional information, if required.

Quantitative outcomes, either extracted or manually calculated from the raw data, were:

• Participation rate: Number of participants divided by number of individuals invited × 100

• Specimen return rate: Number of specimens divided by number of participants × 100

• Testing rate: Number of specimens (number of tests if not reported) divided by number of individuals invited × 100

• CT/NG positivity: Number of positive tests divided by total tests × 100

• Treatment rate: Number of individuals treated divided by number of positive tests × 100

‘Participants’ were defined as individuals who agreed to receive home-collection kits or postal test kits (PTKs) on invitation, requested PTKs through the internet or phone, picked-up PTKs from designated locations, or completed a questionnaire in screening program.

Programs were classified into seven groups (hereafter called program type) based on the recruitment strategy, test-kit and specimen delivery method (Table 1). Programs were defined as population-based if participants were randomly selected from listing of all the individuals (or households) in the target population (e.g. voter register, telephone directory). For the studies presenting weighted CT/NG prevalence estimates, the crude positivity was calculated instead if the required data were available. A frequency analysis was conducted for all the variables. The median, inter-quartile range (IQR) and 95% confidence intervals (CI) were calculated for the rates.

**Table 1:** Description of program type and specimen collection method

**Program type**	**Description**	**Test-kit delivery**	**Specimen return**
**Outreach programs**	Field staff recruited participants at their homes and collected specimens	In-person	In-person
**PTK on invitation acceptance**	People were invited through phone calls and/or letters to receive PTKs, those who agreed were sent a PTK	Post	Post
**PTK along with invitation**	People were sent PTKs along with invitation letters	Post	Post
**PTK with in-person invitation**	People were directly invited to receive a PTK by study personnel	In-person or post	Post
**PTK without invitation**	PTKs were requested through the internet or phone without any direct invitations. Various advertisement strategies were used	Post	Post, drop-off
**PTK with pick-up**	PTKs were picked-up from designated locations (e.g. from boxes at workplace) without direct invitations	Pick-up	Post
**Multiple strategies**	A combination of different strategies was used, but outcomes were not presented separately for each strategy	In-person, post or pick- up	Post

**PTK**, postal test kit.

All the analyses were conducted in STATA 12 (StataCorp, College Station, TX, USA).

## Results

**Figure 1:**
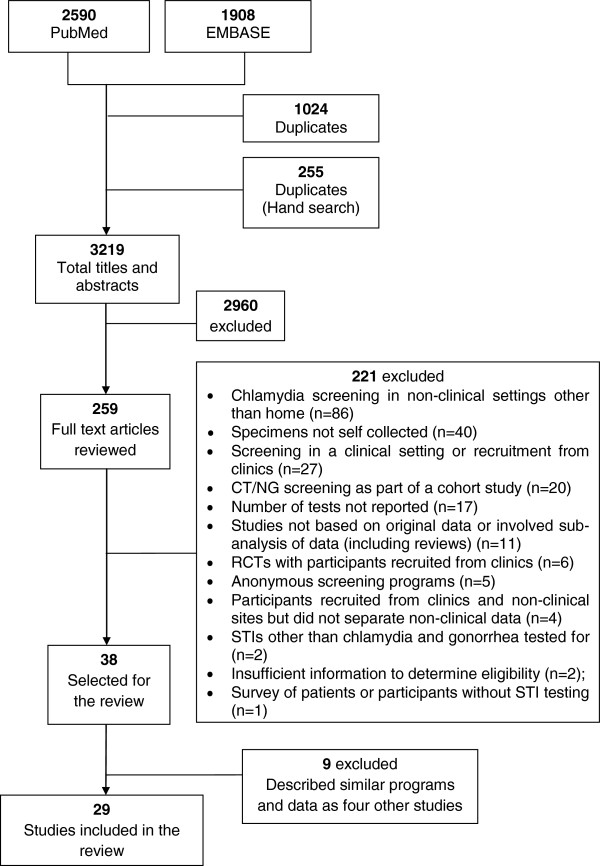
Flow diagram of systematic search strategy.

The initial search led to 3219 unique papers, for which the titles and abstracts were screened (Figure 1). Full-text manuscripts were reviewed for 259 papers, of which 221 were excluded. Of 38 selected, a further nine papers were excluded as they described the same programs as other papers [22-30], but any additional methodology information was extracted. No additional papers were identified from the reference lists.

A total of 29 papers were included in the review [31-59]. One paper described a program established in different settings in two phases [57], the outcomes are reported separately for both phases due to difference in the strategies used. For RCTs with more than one home-testing arm, the data from each arm is presented under the relevant program type [40,43]. The authors of four papers were contacted to collect additional information [41,42,54,58].

### Overview of programs

Programs involved outreach (n=7), PTKs sent on invitation acceptance (n=7), PTKs sent along with invitations (n=5), PTKs requested over the internet or phone without invitations (n=4), PTKs offered by in-person invites (n=4), PTKs picked-up from designated locations (n=3) and the use of two or more of these strategies (n=2) (Table 1). Programs were located in Europe (48%), US (24%), Australia/New Zealand (17%) and other countries (10%). Most programs (69%) targeted both males and females. The specimens consisted of urine only (66%), vaginal swab only (3%), urine for men and vaginal/vulval swab or vaginal flush sample for women (31%).

Across all programs, 81633 tests were conducted (median:550 per program). The overall median participation rate was 68.9% (n=12) and median specimen return rate was 51.4% (n=26). The highest median specimen return rate was in outreach programs (96.5%), followed by programs providing PTKs on invitation acceptance (78.9%), PTKs sent along with invitations (32.9%), PTKs requested without invitation (31.8%), PTKs offered in-person (21.4%), and PTKs picked-up at designated locations (18.6%). The overall median testing rate was 28.8% (n=19), with a CT positivity of 3.6% (n=27) and NG positivity of 0.8% (n=7) (Table 2). Eleven studies reported the treatment rate, with a median of 96% (range: 67-100%).

**Table 2:** Summary of home-based screening outcomes by program type

**Program type**	**Tests**	**Participation rate**	**Specimen return rate**	**Testing rate**	**CT positivity**
	**Total, median, IQR**	**Median, IQR**	**Median, IQR**	**Median, IQR**	**Median, IQR**
**Outreach programs**	13717, 793, 402-3608	83.0%, 82.3-87.8	96.5%, 91.7-99.4	76.1%, 70.7-82.3	2.0%, 1.5-3.6
	(n=7)	(n=5)	(n=6)	(n=5)	(n=5)
**PTK on invitation acceptance**	46225, 657, 105-2580	37.1%, 17.3-65.1	78.9%, 68.3-86.0	18.1%, 12.8-36.2	2.0%, 1.0-4.2
	(n=7)	(n=4)	(n=5)	(n=6)	(n=7)
**PTK along with invitation**	15126,1296, 486-4731		32.9%, 28.8-34.8	28.8%, 23.9-28.8	4.6%, 2.6-5.1
	(n=5)		(n=5)	(n=5)	(n=5)
**PTK without invitation**	2666, 709, 279-1055		31.8%, 26.5-47.4		9.1%, 5.2-12.8
	(n=4)		(n=4)		(n=3)
**PTK with in-person invitation**	341, 37, 5-166	46.4%, 34.7-66.7	21.4%, 19.7-87.5	9.1%, 7.4-58.3	1.5%, 0-9.1
	(n=4)	(n=3)	(n=3)	(n=3)	(n=3)
**PTK with pick-up**	1167, 285, 83-799		18.6%, 12.1-20.2		5.4%, 1.8-9.0
	(n=3)		(n=3)		(n=2)
**Multiple strategies**	2391, 1196, 96-2295				9.2%, 7.3-11.1
	(n=2)				(n=2)
**Overall**	81633, 550, 168-1368	68.9%, 40.6-82.6	51.4%, 22.0-87.5	28.8%, 12.8-65.6	3.6%, 1.7-7.3

**CT**, *Chlamydia trachomatis*; **IQR**, inter-quartile range; **PTK**, postal test kit.

### Findings by program type

#### Outreach

There were seven programs with participant recruitment in homes and immediate collection of specimens. Programs were conducted in the US [31,34,35], UK [32], Barbados [36], Tanzania [33], Pakistan [37], and all were population-based (Table 3).

**Table 3:** Strategies and outcomes of home-based CT and NG screening studies published between Jan 2005-Jan 2011 classified by program type

**Author, year**	**Country**	**Target group, recruitment**	**Sex**	**Tests**	**Participation**	**Specimen return**	**Testing rate**	**CT positive**	**NG positive**
			**Age**		**rate % (95% CI)**	**rate % (95% CI)**	**% (95% CI)**	**% (95% CI)**	**%(95% CI)**
**Outreach Programs (n=7)**									
Datta, 2007 [31]	US	Screening within a national survey^A^	M/F;	6632	83.0	91.7	76.1	3.6%	0.5%
			14-39		(82.2-83.8)	(91.1-92.4)	(75.2-77.0)	(3.2-4.1)	(0.4-0.8)
McCadden, 2005 [32]	Brittain (UK)	Randomly selected (national survey^B^)	M/F;	3608	71.1	99.4	70.7	2.0%	
			18-44		(69.8-72.3)	(99.1-99.7)	(69.4-71.9)	(1.6-2.5)	
Ghebremichael, 2009 [33]	Tanzania	Randomly selected households	F;	1439	92.1	71.3	65.6	1.5%	0.2%
			20-24		(90.9-93.2)	(69.2-73.2)	(63.6-67.6)	(1.0-2.3)	(0.0-0.6)
Forhan, 2009 [34]	US	Screening within a national survey ^A^	F;	793		94.6		3.9 ^C^	
			14-19			(92.9-96.1)			
Jennings, 2010 [35]	US	Randomly selected households; Monetary incentives	M/F;	587	87.8	98.3	86.4		
			15-24		(85.1-90.2)	(97.0-99.2)	(83.6-88.9)		
Adams, 2008 [36]	Barbados	Randomly selected (voter’s register)	M/F;	402^D^	82.3%	100	82.3	11.3%	1.8%
			18-35		(78.6-85.5)	(99.1-100)	(78.6-85.5)	(8.4-14.9)	(0.7-3.6)
Mir, 2009 [37]	Pakistan	Randomly selected households in a survey	M;	256				0.0%	0.8%
			16-45						(0.1-2.8)
**Programs with PTKs sent on invitation acceptance (n=7)**									
*Van Bergen, 2010 [38]	Nether-lands	Participants form population register, PTKs requested through internet; Reminders	M/F;	41638	20.2	78.9	16.0	4.2%	
			16-29		(20.1-20.4)	(78.6-79.3)	(15.8-16.1)	(4.0-4.4)	
Goulet, 2010 [39]	France	Randomly selected (national survey); Reminders	M/F;	2580	76.3	68.3	52.0	1.7%	
			18-44		(75.0-77.4)	(66.7-69.7)	(50.6-53.4)	(1.2-2.2)	
*Anderson, 2010 [40]	Denmark	Randomly selected (county health service register)	M/F;	912			20.3	7.0%	
			22-24				(19.1-21.5)	(5.4-8.9)	
Hocking, 2006 [41]	Australia	Random household sample (telephone directory)	F;	657	53.9 ^E^	67.1	36.2^E^	0.9%	
			18-35		(51.6-56.2)	(64.1-70.0)	(33.9-38.4)	(0.3-2.0)	
Domeika, 2007 [42]	Sweden	Randomly selected (population register, student register); Advertised	M/F;	247	14.5	88.2	12.8	2.0%	
			19-23		(12.9-16.1)	(83.8-91.7)	(11.3-14.3)	(0.7-4.7)	
*Scholes, 2007 [43]	US	Participants from enrollees in a managed care plan; Reminders	M;	105			3.6	1.0%	
			21-25				(2.9-4.3)	(0.0-5.2)	
Eggleston, 2005 [44]	US	Telephone accessible households; Monetary incentive; Reminders	M/F;	86		86.0		2.3%	0.0%
			18-35			(77.6-92.1)		(0.3-8.1)	
**Programs with PTKs sent along with invitation (n=5)**									
Van Bergen, 2005 [45]	Nether-lands	Randomly selected (civilian registry); Reminders	M/F;	8383		40.3**	39.9	2.0%	
			15-29			(39.7-41.0)	(39.3-40.6)	(1.7-2.3)	
Low, 2007 [46]	England	Randomly selected (general practice lists); Reminders	M/F;	4731		32.9**	23.9	4.6%	
			16-39			(32.1-33.7)	(23.3-24.5)	(4.0-5.3)	
*Anderson, 2010 [40]	Denmark	Randomly selected (county health service register)	M/F;	1296		28.8	28.8	6.2%	
			22-24			(27.5-30.1)	(27.5-30.1)	(4.9-7.6)	
Uuskula, 2008 [47]	Estonia	Randomly selected (population registry)	M/F;	486		34.8**	28.8	5.1%	
			18-35			(32.3-37.4)	(26.7-31.0)	(3.4-7.5)	
*Scholes, 2007 [43]	US	Participants from enrollees in a managed care plan; Reminders	M;	230		7.8 (6.9-8.9)	7.8	2.6%	
			21-25				(6.9-8.9)	(1.0-5.6)	
**PTKs without invitation programs (n=4)**									
Gaydos, 2009 [48]	US	PTKs requested through the internet; Advertised	F;	1203		32.4		9.1%	1.3%
			>=14			(30.9-33.9)		(7.5-10.8)	(0.8-2.2)
Novak, 2006 [49]	Sweden	PTKs requested through the internet; Advertised	M/F	906		62.5		5.2%	
						(59.9-65.0)		(3.8-6.8)	
Chai, 2010 [50]	US	PTKs requested through the internet; Advertised	M;	512		31.1		12.8	0.8%
			>=14			(28.9-33.4)		(10.0-16.0)	(0.02-2.0)
Martin, 2009 [51]	Australia	PTKs requested through the internet/phone, specimens dropped-off; Advertised	M/F;	45		22.0			
			16-24			(16.5-28.2)			
**PTKs with in-person invitation programs (n=4)**									
Brabin, 2009 [52]	England	PTKs offered to women requesting EHC at pharmacies	F;	264	46.4	19.7	9.1	9.1%	
			<=24		(44.6-48.3)	(17.6-21.9)	(8.1-10.2)	(5.9-13.2)	
Sacks-Davis, 2010 [53]	Australia	People at a music festival invited to receive PTKs; Non-monetary incentive; Reminders	M/F;	67	34.7	21.4	7.4	1.5%	
			16-29		(31.6-37.9)	(17.0-26.4)	(5.4-9.3)	(0.0-8.0)	
Dabrera, 2010 [54]	England	PTKs offered to women requesting EHC at pharmacies	F;	7	66.7	87.5	58.3		
			<=21		(34.9-90.1)	(47.3-99.7)	(27.7-84.8)		
Rose, 2010 [55]	New Zealand	PTKs offered to general practice clients to pass to their social contacts	M/F	3				0.0%	
**PTKs with pick-up programs (n=3)**									
Davison, 2007 [56]	Scotland	PTKs picked-up from GUM clinic, youth service, family planning clinic etc.	M/F	799		20.2		9.0%	
						(18.9-21.5)		(7.1-11.2)	
MHF, 2005 [57]	England	PTKs (pick-up) were available to employees at 6 workplaces; Advertised	M;	285 ^F^		12.1		1.8%	
			<=30			(10.8-13.5)		(0.6-4.0)	
MHF, 2005 [57]	England	PTKs available for pick-up at 5 non-clinical sites	M;	83		18.6^G^			
			<=30			(15.1-22.5)			
**Programs with multiple strategies (n=2)**									
Williamson, 2007 [58]	Scotland	PTKs distributed or picked-up at various locations	M/F;	2295^H^				11.1%	
			13-25					(9.9-12.5)	
Buhrer-Skinner, 2009 [59]	Australia	PTKs requested through internet/phone or picked-up at different locations; Advertised	M/F;	100				7.3%	
			16-25					(3.0-14.4)	

**Definitions and abbreviations: Participation rate**, participants divided by number invited × 100; **Specimen return rate**, number of specimens (or tests) divided by participants × 100; **Testing rate,** number of specimens divided by number invited × 100. **CT**, *Chlamydia trachomatis*; **NG**, *Neisseria gonorrhoeae*; **M**, Male; **F**, Female; **US**, United States; **UK**, United Kingdom; **PTK**, postal test kit; **GUM**, genitourinary medicine; **MHF,** Men’s Health Forum.

* Randomized Controlled Trial (RCT) ** calculated among those who received PTKs (excluded undelivered kits).

^**A**^ National Health and Nutrition Examination Survey (NHANES); ^**B**^ National Survey of Sexual Attitudes and Lifestyles (Natsal); ^**C**^ weighted CT prevalence; ^**D**^ 397 valid tests; ^**E**^ calculated among 1817 eligible contactable participants after excluding 6555 ineligible and 2629 un-contactable out of 11001 households sampled; ^**F**^ although the program was targeted at male employees, some of the specimens were returned by female employees; ^**G**^ specimen return rates for individual locations: Agricultural college, 41.0% (41 tests); Factory, 36.0% (9); Satellite college of university, 14.3% (4); Military Police training center 13.6% (12); Post-16 college 8.3% (17); ^**H**^ 20% of returned kits were distributed from clinics and 10% were picked-up form university.

Across these programs, 13717 tests were conducted (median: 793). The median participation rate was 83.0% (n=5), with a specimen return rate of 96.5% (n=6) and testing rate of 76.1% (n=5). The median CT positivity was 2.0% (n=5) and NG positivity was 0.7% (n=4) (Table 2). Four programs encouraged participants to contact the staff for test results [31-34]. One program reported a 67% treatment rate (n=49), with 24 of 28 traced sexual contacts (86%) also treated [32].

#### PTK on invitation acceptance

In these seven programs, people were invited to receive PTKs through phone calls (n=1), letters (n=4), or letters and phone calls (n=2). Programs were conducted in the US [43,44], Australia [41], France [39], Denmark [40], Sweden [42] and the Netherlands [38] (Table 3). Six population-based programs randomly selected participants from population, health service or student registers [38,40,42], telephone directories [41,44], and from a national survey [39]. Another study was conducted at a health care plan [43]. In one program, participants requested PTKs through a website after receiving an invitation letter [38]. In this program, in less prevalent areas, only individuals with risk-scores above a certain level could request PTKs after mandatory online risk-assessment [38]. In other programs, people requested a PTK via phone or by returning prepaid reply cards.

Across these programs, 46225 tests were conducted (median: 657). The median participation rate was 37.1% (n=4), specimen return rate was 78.9% (n=5) and testing rate was 18.8% (n=6). The median CT positivity was 2.0% (n=7) (Table 2). Reminders were used in four programs, which accounted for 41% of specimens in one program (1-5 phone calls, new PTK) [44], 39% of specimens in the second program (two emails) [38] and an increased specimen return rate from 29% to 68% in the third program (phone call, two invitation letters, new PTK) [39]. The fourth program did not report the effect of reminders (one letter) [43].

Participants were either notified of test results [39-41,43,44], or could access results online [38,42]. One program had a 100% treatment rate (n=1) [43], a second program reported 84% treatment rate (n=36) with 81% of contacts (n=22) also treated [39], while a third program reported 89% treatment rate (n=892) among 43% of positive individuals who completed a questionnaire [38]. The cost of one telephone survey and test-kit delivery was US$250-300 in one program [44], and the cost of diagnosing one infection was SEK14000 (US$2020) in another program [42].

#### PTK sent along with invitation

In these five programs, PTKs were sent to participants’ homes along with invitation letters. Programs were conducted in the US [43], England [46], Denmark [40], Estonia [47] and the Netherlands [45] (Table 3). Four population-based programs randomly selected participants from the population register [45,47], health service register [40] and general practice lists [46], while another program selected participants from a health-care plan [43].

A total of 15126 tests were conducted (median: 4731). The median specimen return rate was 32.9% (n=5) and testing rate was 28.8% (n=5) (Table 2). The median CT positivity was 4.6% (n=3) (Table 2). Three programs used reminders, which accounted for 10% of specimens in one program (letter, phone call, home visit/flagging patient records) [46], 18% of specimens in the second program (letter or new PTK) [45], while the third program did not report the effect of reminders [43].

Test results were notified to participants in all the programs. The treatment rate was reported in two programs, 100% (n=6) in one program [43] and 91% (n=150) in the second program with 49% of partners (n=86) also treated [45]. The operational cost of one program was £14.65 (US$23) per invitation and £21.47 (US$34) per individual screened [46].

#### PTK without invitation

These four programs in the US [48,50], Australia [51] and Sweden [49] used internet or telephone to request PTKs without any direct invitations (Table 3). These programs used several advertising strategies for promotion. The specimens were returned by post, except one program which required specimens to be dropped-off in boxes at selected locations [51]. In one program, in addition to the internet, PTKs could be picked-up at community locations but this method was discontinued due to a poor response rate [48].

A total of 2666 tests were conducted (median: 709) with the median specimen return rate of 31.8% (n=4). The median CT positivity was 9.1% (n=3) (Table 2). Test results were accessible to participants in all the programs. The treatment rate in three programs was 97% (n=105) [48], 99% (n=105) [50] and 100% (n=47) [49].

#### PTK with in-person invitation

Four programs involved study personnel directly inviting people to receive PTKs and were based in England [52,54], Australia [53] and New Zealand [55] (Table 3). Two programs were in pharmacies [52,54], one in general practice [55] and one at a music festival [53]. A total of 341 tests were conducted (median: 37). The median participation rate was 46.4% (n=3), specimen return rate was 21.4% (n=3) and testing rate was 9.1% (n=3). The median CT positivity was 1.5% (n=3) (Table 2). SMS reminders were used in one program [53]. Test results were notified to participants in two programs [53,55], and one program reported a 92% treatment rate (n=22) [52].

#### PTK with pick-up

In these three programs, PTKs were available for pick-up from designated locations [56,57] (Table 3). In the Men and Chlamydia Project (M&CP) [57], employees of six workplaces in England picked-up PTKs from boxes in gents’ toilets, locker rooms and restrooms. The second program, an extension of M&CP [57], included five non-clinical sites. In the third program in Scotland [56], PTKs were picked-up from the door of a genitourinary medicine clinic, a youth service, family planning and other sources.

Across these programs, 1167 tests were conducted (median: 285). The median specimen return rate was 18.6% (n=3) and the median CT positivity was 5.4% (n=2) (Table 2). Results were notified to participants in the M&CP only and the treatment rate was 100% (n=5) [57]. The cost per test, excluding operational cost, in M&CP was £8.36 (US$13) and the cost per diagnosed infection was £695 (US$1079).

#### Multiple strategies

Two programs used more than one strategy for screening, but did not report the outcomes separately (Tables 2, 3). In an Australian study [59], PTKs were picked-up from pharmacies, tertiary education facilities, community groups and sports clubs, or requested through the internet and telephone. Only first 100 kits were analyzed and the treatment rate was 100% (n=7) [59]. In a Scottish study [58], PTKs were picked-up from boxes at commercial venues including pharmacies, young peoples’ drop-ins and record stores, or distributed at drop-in venues, with 2295 specimens submitted for screening.

## Discussion

Home-based CT and NG screening programs have been conducted in many countries with a range of strategies for recruitment, test-kit delivery and specimen-collection. A number of programs were population-based, most used PTKs and some involved the use of internet for requesting test-kits. The overall median specimen return rate for programs included in this review was 51.4%, the median testing rate was 28.8% and the median CT positivity was 3.6%.

The key strength of this systematic review is the large number and range of home-based screening programs from a number of countries, which allowed examination of different strategies and outcomes. We used standardised definitions for the key outcomes to allow comparisons within and across program types. However, there are a few limitations. Firstly, we did not search the grey literature and thus may not have included other relevant unpublished programs. Secondly, we were unable to report the key outcomes for all programs, such as participation and testing rates, due to design of the programs or necessary data not being reported. Thirdly, any comparison of CT/NG positivity across the programs is limited by different target populations studied and known prevalence in the underlying populations.

This review included a number of outreach programs. The high specimen return rates in these programs indicate that majority of people agreed to provide specimens when approached in their homes. In these programs, specimen-collection was integrated into ongoing national [31,32,34] and population-based surveys [33,35-37], and hence incurred no significant additional cost and human resources. The downside of this approach is that such surveys are often conducted infrequently and are therefore more suitable for estimating the prevalence than being a method for ongoing screening.

The use of mailed specimens appears to have increased in popularity in recent years as most programs in this review used PTKs for screening. The recruitment strategies in these programs have varied considerably. Programs with PTKs mailed alongside an invitation have been implemented in a few European countries, but the specimen return rates were low. Programs which sent PTKs on invitation acceptance, on the other hand, were associated with relatively higher specimen return rates. Some PTK programs required the test-kits to be collected or offered at specific physical locations. These were associated with relatively less people being tested as well as low specimen return rates.

A few PTK programs in the review required the test-kits to be requested through the internet. PTKs requested in this way appear to be a logistically feasible strategy for establishing large population-based screening programs, as demonstrated by the initial results of a trial in the Netherlands [38]. The three year results of this register-based yearly CT screening program published recently demonstrate no significant decrease in CT positivity in the target population after three screening rounds, with the testing rates declining in each round [60]. However, among people who were screened in all three years, the positivity dropped from 5.9% to 2.9% [60]. The number of individuals screened in the first round (n=41638) was greater than in any other program in the review [38]. The internet can be useful for selective screening of high-risk people through the completion of online risk-assessment questionnaires, as done in less CT prevalent areas in the Netherlands [38]. Other internet programs in the review did not involve direct invitations and relied on people actively seeking PTKs after programs were advertised [48-50]. Relatively few people were tested by this approach but CT positivity was higher, which may be due to people self-selecting on the basis of their risk. The internet also provides an opportunity to deliver test results online in a confidential manner at the individual’s convenience [38,42,49].

The use of reminders has shown to improve the specimen return rates [38,39,44-46]. However, reminder implementation on a large scale, along with notification of results and contact-tracing, can be resource intensive [61] and may require comprehensive registries. There may be other logistic challenges in establishing PTK programs, such as collection and transport of specimens. Clinical specimens must comply with international and national packaging requirements for transport [62]. The current three-layered packaging system for infectious substances often results in large packages (at least one surface with minimum 100×100 mm dimension) [63], which may require delivery and collection at the post-office or through a courier, and thus costly to transport [62]. However, a recently developed sponge-based urine-collection device called UriSwab (Copan Diagnostics, Inc.) holds a small amount of urine after being held in the urine flow or dipped in a specimen cup. UriSwab is easy to transport, has shown good performance in detecting CT/NG infections and can potentially facilitate the establishment of PTK programs [64]. Other considerations in home-based screening programs may include the issues of privacy and confidentiality, such as ensuring communication of results, treatment and contact tracing in a confidential manner and in-line with the individual’s preference [61,62].

Home-based testing can potentially reduce individual screening costs by avoiding clinic fees as well as the indirect costs, such as time off work and transportation [65]. However, there is no conclusive evidence of the cost-effectiveness of home-based screening over routine clinic-based screening. Four programs in this review provided cost information in association with home-based screening [42,44,46,57]. Only Low and colleagues reported full operational cost of a PTK screening program [46], while Domeika et al. compared the cost of home-based screening in their study with routine screening and reported the former to be about five times higher [42]. One RCT compared the cost of home and clinic-based screening and found that home-screening provided a cost-saving ($25 per test in home vs. $111 in clinic after including direct and indirect costs) [65], but that the cost-saving was not seen when the results were restricted to asymptomatic tests. It is thus important for future programs to focus on estimating operational costs in relation to the screening outcomes to establish the cost-effectiveness of home-based screening.

## Conclusions

This systematic review shows that home-based screening programs have been conducted in various countries and have utilised a variety of strategies. Home-based testing with self-collected specimens appears to be an acceptable and logistically feasible method for CT and NG screening outside of clinics. However, economic evaluation of large-scale home screening programs is warranted to assess their cost-effectiveness in the real-world scenario. The recruitment and specimen-collection strategies adopted for home-based screening and their potential impact on the outcomes need to be carefully considered. A pilot program to assess the feasibility of screening in the local context would be highly recommended before embarking on a large-scale program. Ongoing assessment of the outcomes and subsequent modification of strategies should be considered to improve the effectiveness of screening programs.

## Abbreviations

CT: *Chlamydia trachomatis*; NG: *Neisseria gonorrhoeae*; NAAT: Nucleic acid amplification test; PTK: Postal test kit; STI: Sexually transmissible infection; US: United States; UK: United Kingdom; RCT: Randomized controlled trial; CI: Confidence interval; IQR: Inter-quartile range; M&CP: Men and Chlamydia Project.

## Competing interests

The authors declare that they have no competing interests. No funding was received by the authors for this systematic review.

## Authors’ contributions

RJG and MSJ conceptualized the study and developed the search strategy. MSJ conducted the search. MSJ and RJG reviewed the papers with input from HMB, JSH and JW and extracted the data. HW performed the statistical analysis. MSJ and RJG drafted the manuscript. All the authors read and approved the final manuscript.

## Pre-publication history

The pre-publication history for this paper can be accessed here:

http://www.biomedcentral.com/1471-2458/13/189/prepub
